# Oxygen saturation and heart rate in healthy term and late preterm infants with delayed cord clamping

**DOI:** 10.1038/s41390-021-01805-y

**Published:** 2022-01-07

**Authors:** Inmaculada Lara-Cantón, Shiraz Badurdeen, Janneke Dekker, Peter Davis, Calum Roberts, Arjan te Pas, Máximo Vento

**Affiliations:** 1 Neonatal Research Group, Health Research Institute and University and Polytechnic Hospital La Fe, Valencia, Spain; 2 Newborn Research Center and Neonatal Services, The Royal Women´s Hospital, Melbourne, VIC Australia; 3 Division of Neonatology, Department of Paediatrics, Leiden University Medical Centre, Leiden, The Netherlands; 4 Department of Paediatrics, Monash University, Clayton, VIC Australia

## Abstract

**Abstract:**

Blood oxygen in the fetus is substantially lower than in the newborn infant. In the minutes after birth, arterial oxygen saturation rises from around 50–60% to 90–95%. Initial respiratory efforts generate negative trans-thoracic pressures that drive liquid from the airways into the lung interstitium facilitating lung aeration, blood oxygenation, and pulmonary artery vasodilatation. Consequently, intra- (foramen ovale) and extra-cardiac (ductus arteriosus) shunting changes and the sequential circulation switches to a parallel pulmonary and systemic circulation. Delaying cord clamping preserves blood flow through the ascending vena cava, thus increasing right and left ventricular preload. Recently published reference ranges have suggested that delayed cord clamping positively influenced the fetal-to-neonatal transition. Oxygen saturation in babies with delayed cord clamping plateaus significantly earlier to values of 85–90% than in babies with immediate cord clamping. Delayed cord clamping may also contribute to fewer episodes of brady-or-tachycardia in the first minutes after birth, but data from randomized trials are awaited.

**Impact:**

- Delaying cord clamping during fetal to neonatal transition contributes to a significantly earlier plateauing of oxygen saturation and fewer episodes of brady-and/or-tachycardia in the first minutes after birth.
- We provide updated information regarding the changes in SpO_2_ and HR during postnatal adaptation of term and late preterm infants receiving delayed compared with immediate cord clamping.
- Nomograms in newborn infants with delayed cord clamping will provide valuable reference ranges to establish target SpO_2_ and HR in the first minutes after birth.

## Introduction

To objectively guide resuscitation, monitoring of arterial oxygen saturation (SpO_2_) and heart rate (HR) using pulse oximetry has become standard of care.^1–3^ In 2010, Dawson et al.^4,5^, merging three databases from the Royal Women’s Hospital (Melbourne) and the University and Polytechnic Hospital La Fe (Valencia), established a reference range for SpO_2_ and HR. In a prospective observational study, a pulse oximeter sensor was placed on the right hand or wrist (preductal), and minute-by-minute SpO_2_ and HR were measured in 308 term and 160 preterm infants during the first 10 min after birth. Percentile charts for SpO_2_ and HR in term and preterm infants were constructed using these data.^4,5^ In 2015, the American Heart Association (AHA) recommended the following target ranges for SpO_2_: at 1 min 60–65%, at 2 min 65–70%, at 3 min 70–75%, at 4 min 75–80%, at 5 min 80–85%, and at 10 min 85–95%, keeping HR > 100 beats per minute (bpm) during postnatal stabilization, and these target ranges have been recommended to date.^6^

Recently, international guidelines have strongly recommended delaying cord clamping for 30–60 s.^7^ Delayed cord clamping (DCC) as opposed to immediate cord clamping (ICC) preserves a steady blood flow from the placenta to the newly born circulation enhancing ventricular preload and will contribute to a more stable hemodynamic transition when lung aeration occurs in before cord clamping.^8^

The aim of this review article is to provide readers with updated information regarding the changes in SpO_2_ and HR during postnatal adaptation of term and late preterm infants receiving DCC.

## Intrauterine oxygenation

The partial pressure of oxygen gradient between maternal, placental, and fetal blood is the driving force that regulates fetal oxygen supply. Both placenta and embryo grow and differentiate in a relatively low oxygen environment during the first trimester. The blood flow to the intervillous space (IVS) becomes fully established at the 11–12th weeks of gestation. Coincidentally, oxygen tension within the IVS abruptly rises from 18 mmHg in the eighth week to 60 mmHg at 12 weeks.^9^ In the second half of pregnancy, IVS partial pressure of oxygen slowly decreases from around 60 mmHg in the 12th week of gestation to 45 mmHg at term assuring a steady-state oxygenation of the fetus.^10^ The limited supply of oxygen to the fetus requires adaptive changes in the placental structure together with changes in fetal metabolism. The relatively low metabolic rate of the fetus combined with thermoregulation provided by the mother leads to an important reduction in fetal oxygen consumption relative to the newborn infant.^11^ Another important factor contributing to adequate fetal oxygenation is the sustained increase in fetal hemoglobin concentration throughout gestation. Fetal hemoglobin has high affinity for oxygen at the placental level. Moreover, due to fetal hemoglobin and a low level of 2,3 diphosphoglycerate, the oxygen dissociation curve shifts to the left, which results in a greater release in oxygen at a lower arterial PO_2_ compared to adult hemoglobin, facilitating the liberation of oxygen into peripheral tissues.^12^ However, the most important mechanism that ensures that fetal oxygenation is kept within physiologic limits, comparable to those of the newborn infant, is the high perfusion of the fetal organs. The high fetal cardiac output of 250–300 mL/kg/min is achieved due to the fast HR and the central shunting that allows the fetal ventricles to work in parallel, rather than in series as in the adult-type circulation. Central shunting redirects oxygenated blood to the left ventricle for preferential distribution to the brain and myocardium.^13^

## Oxygenation during fetal-to-neonatal transition

Oxygenation plays an important role in the physiological changes needed for a successful fetal-to-neonatal transition. In uncompromised and adequately breathing infants, SpO_2_ increases from 50–60% in the fetus to 90–95% in the first minutes after birth.^4^ This increase is needed to match the rise in metabolic rate that comes with the physiological adaptation to extra-uterine life (thermoregulation, breathing effort).^14^ The activation of non-shivering thermogenesis attained through the oxidation of brown fat requires increased oxygen supply. It has been demonstrated that hypoxia acts as a suppressor of this non-shivering thermogenesis and infants struggle to maintain thermoregulation in a cold environment.^14^

Fetal breathing movements are inhibited by low oxygen levels. It has been shown that hypoxia directly inhibits neural input to the respiratory center from a region located in the upper lateral pons, at the level of the middle cerebral peduncle in the region of the principle sensory and motor nuclei of the trigeminal nerve.^15^ During apnea in the fetus, glottis adduction results in a restriction of loss of airway liquid and is therefore vital for lung development.^16^ However, this adduction of the glottis during apnea also persists after birth, thereby restricting the aeration of the lung using non-invasive techniques.^17,18^ A temporal change in O_2_ sensitivity takes place after birth,^19^ and while most preterm infants breathe,^20^ it is not known when the switch from respiratory suppression to stimulation occurs in response to hypoxia. Large inspiratory efforts are needed immediately at birth to establish lung liquid clearance and lung aeration. Hypoxia may interfere with these processes as it leads to depressed or absent breathing efforts, particularly in preterm infants.^20,21^ On the other hand, hyperoxia also seems to inhibit the chemoreceptors as the onset of breathing was delayed in asphyxiated animals and infants receiving 100% oxygen.^22^

Oxygen is known as a potent vasodilator, and it is commonly assumed that oxygenation of the lungs is predominantly responsible for the physiological fall in pulmonary vascular resistance at birth. However, it has recently been demonstrated in a lamb model that the lung liquid clearance and aeration established with ventilation is the primary trigger for a prompt decrease in pulmonary vascular resistance.^23,24^ Although the onset of ventilation seems to be “the master switch”, oxygen seems to be additive to this as a “mediator”.

## Introduction of pulse oximetry monitoring in the delivery room

For many years, evaluation of the condition of the newborn infant in the first minutes of life was based on clinical signs including HR and color. HR was considered the most objective measure of a newborn infant’s condition and thresholds (60 and 100 bpm) became widely accepted indications for the commencement of interventions including respiratory support and cardiac compressions. Studies of HR assessment by auscultation and umbilical cord palpation revealed that these methods were imprecise, and systematically underestimated HR.^25^ Similarly, when asked to assess color, clinicians of all levels of experience could not agree when an infant became pink.^26^ In 2003, John Kattwinkel^27^ suggested in a commentary accompanying an air vs 100% oxygen trial that rather than choosing a fixed oxygen concentration to use throughout a resuscitation, *“*perhaps we should be aiming to restore normoxia quickly and to achieve normal levels of blood oxygen throughout and beyond the resuscitation process. More aggressive use of the pulse oximeter in the delivery setting may facilitate achieving this goal”. Pulse oximetry offers the additional benefit of being able to display HR in real time without clinicians having to shift their focus from the provision of resuscitation.

Kattwinkel’s paradigm-shifting suggestion led to several questions. Although used for many years in the neonatal intensive care unit, pulse oximetry was untested in the delivery room (DR). Questions at the time were whether it would provide reliable readings when applied to a wet, often poorly perfused newborn infant in need of resuscitation, and if it did, would it provide oxygen saturation and HR values early enough in the infant’s course to be clinically useful?

O’Donnell et al.^28^ showed that pulse oximetry provided data in over 90% of babies in the DR, and within 90 s of life in 90%. They also demonstrated that applying the pulse oximeter probe to the infant before connecting to the oximeter resulted in quickest acquisition of accurate HR.^29^ In the first minutes of life, right-to-left ductal shunting predominates and therefore SpO_2_ is higher in the preductal circulation than in the postductal circulation.^30^ Therefore, it is recommended that the probe should be applied to the right hand or wrist to ensure that preductal oxygen saturation levels which reflect cerebral values are displayed. Kamlin et al.^31^ showed that the pulse oximeter had reasonable sensitivity (89%) and excellent specificity (99%) in detecting HRs less than 100 bpm.^31^

For clinicians used to targeting oxygen therapy to achieve saturation levels in the 90 s for babies in the neonatal intensive care unit, it quickly became apparent that the concept of “normoxia” suggested by Kattwinkel needed a new set of target values to be clinically useful.

## The Dawson nomogram

In 2010, Dawson et al.^4^^[,5^ published two seminal studies aimed at providing reference ranges for SpO_2_ and HR in transitioning infants.^4,5^ Although this work was preceded by other attempts to describe normal ranges,^32–34^ Dawson’s smoothed percentile charts that included data collected every 2 s rapidly became the most widely used. The SpO_2_ nomograms provided a reference against which an infant’s oxygenation status could be objectively compared.

Dawson’s nomograms were derived from a prospective cohort of 468 infants (*n* = 39 born at <32 weeks PMA, *n* = 121 at 32–36 weeks PMA, *n* = 308 at ≥37 weeks PMA) at two tertiary centers. As was usual care at the time, infants received ICC. Infants who received supplemental oxygen or ventilation were excluded.^4,5^ Informed by the results, resuscitation guidelines have generally used approximations of the 25th percentile of Dawson’s nomogram as the lower acceptable SpO_2_ level, broadly recommending preductal SpO_2_ levels of >65% by 2 min and >80% by 5 min.^6,35,36^ Of note, Dawson et al.^4^ found that preterm infants on average had median SpO_2_ levels 3–8% lower than that of term infants. Infants born by cesarean section had median SpO_2_ levels approximately 10% lower than those of vaginally born infants in the first 5 min.

The nomograms quickly evolved from descriptive reference ranges to recommended SpO_2_ targets. Clinicians caring for an infant at the lower end of the distribution for oxygen saturation were provided with a trigger SpO_2_ level at which to commence oxygen therapy, and targets to titrate that therapy over the first 10 min after birth. There are limited data on the consequences of the broad strategy of titrating oxygen to mimic SpO_2_ levels of infants who transition without intervention.^37^ The practice has likely reduced the incidence of untreated hypoxia in the delivery room and associated mortality in preterm infants.^38^ However, an infant who starts at and progresses along the 10th centile is likely to receive oxygen therapy, and it is not known whether this is the optimal strategy.

The HR nomograms derived from infants in the same cohort have attracted less attention.^5^ DR practice has continued to focus on a dichotomous threshold of <100 bpm as a trigger for intervention. Dawson’s nomograms suggested that 50% of infants have HRs below this threshold at 1 min after birth and cautioned against intervention if the infant appeared clinically well. This observation has contributed to an ongoing body of work aimed at establishing whether delaying cord clamping would provide greater physiological stability during fetal-to-neonatal transition.^39,40^ Additional comparisons of infant HR measured concurrently using 3-lead electrocardiogram and pulse oximetry have highlighted discrepancies in the first 2 min after birth.^41^ This discrepancy, not apparent when using the Nelcor oximeter,^42^ raises the possibility of artifact contributing to measurements of low HR using the Masimo pulse oximeter.

## Postnatal oxygenation with DCC

When considering oxygenation at birth, it seems logical to defer cord clamping, which removes the placenta as a gas exchange organ, until the lungs have been aerated and are able to provide gas exchange. Deferring cord clamping until after ventilation (physiological based cord clamping, PBCC) sustains preload and cardiac output and avoids the large disturbances in systemic and cerebral circulation associated with ICC.^37,43^ These studies, conducted in preterm lambs, demonstrated that a substantial drop in SpO_2_ occurred after ICC, with a subsequent tachycardia in the 3 min after ventilation onset, whereas when PBCC was applied, these values remained stable.^37,43^ This was later confirmed in a clinical setting in preterm infants.^44^

Early published clinical studies have focused on data of term infants. The first prospective cohort included 109 term infants receiving DCC of ≥1 min and immediate skin-to-skin care after low-risk births. Pulse oximetry was used to record SpO2 and HR.^45^ In comparison with the ICC reference ranges, these infants had significantly higher SpO_2_ values in the first 3 min of life, and lower values (but with median SpO2 90–95%) between 5 and 10 min. The DCC infants had lower HR than the ICC reference ranges from 2 to 10 min of age: the largest difference in median HR was at 2 min (85 vs 160 bpm) with a difference of 18 bpm or less thereafter. The DCC infants were less likely to have tachycardia of >180 bpm (2.6% vs 19.3%) and more likely to have bradycardia of <80 bpm (6.5% vs 4.8%) during the first 10 min. The authors noted that the high rate of immediate skin-to-skin care and breastfeeding in the DCC cohort may have contributed.^45^

Two RCTs have included term infants born by elective cesarean section, allocating them to either ICC, or DCC of at least 60 s. Cavallin et al.^46^ included 80 infants and did not identify a significant difference in either SpO_2_ or HR in the first 10 min after birth. Their sample size was not powered for these outcomes, and data analysis excluded the first 3 min after birth, where differences may have been more pronounced. Both groups had median SpO2 > 80% and median HR > 160 at 3 min. De Bernardo et al.^47^ included 132 infants born at 37–42 weeks, finding similar SpO2 and HR values, but again the times of assessment (5 and 10 min) excluded the first minutes after birth.

**Fig. 1:**
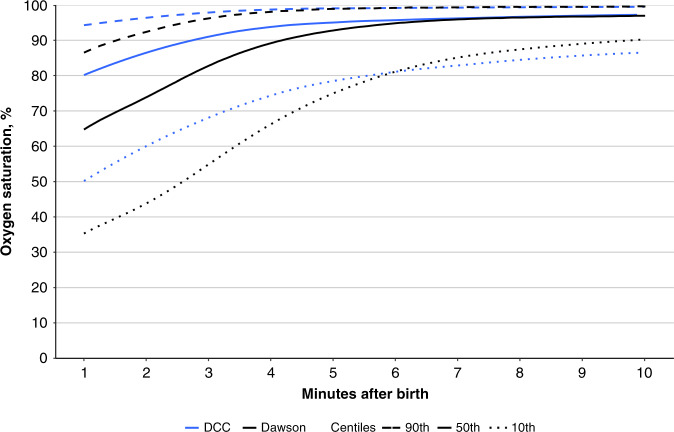
Comparison of Dawson’s with delayed cord clamping (DCC) nomogram for arterial oxygen saturation (SpO_2_). Comparison of the 10th, 50th, and 90th percentiles for SpO_2_ measured with preductal pulse oximetry between the reference range in Dawson’s nomogram for oxygen saturation (ref. ^4^) and babies with delayed cord clamping (DCC) for >60 s in the study by Padilla-Sánchez et al.^49^ reproduced with permission of *The Journal of Pediatrics*.

**Fig. 2:**
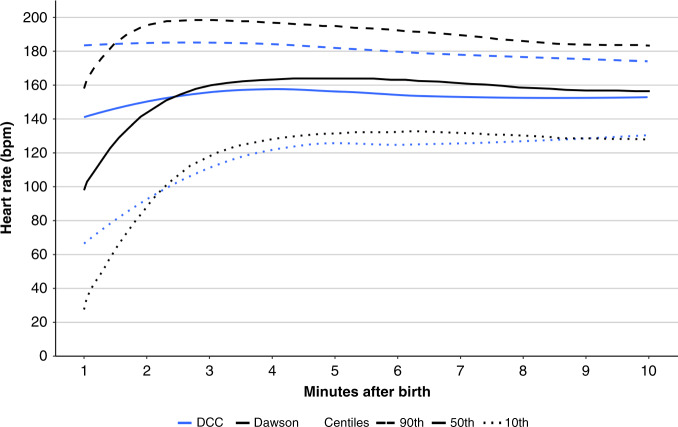
Comparison of Dawson’s with delayed cord clamping (DCC) nomogram for heart rate (HH). Comparison of the 10th, 50th, and 90th percentiles for heart rate (HR) measured with preductal pulse oximetry between the reference range in Dawson’s nomogram for heart rate (ref. ^5^) and babies with delayed cord clamping (DCC) for >60 s in the study by Padilla-Sánchez et al.^49^ reproduced with permission of *The Journal of Pediatrics*.

Padilla-Sanchez et al.^48^ published oximetry data from a cohort of 282 infants of ≥37 weeks’ gestation born vaginally, who did not receive resuscitation and underwent DCC for ≥1 min (mean 110 s). They compared their findings with the Dawson nomograms for vaginally born infants, noting that the DCC infants had significantly higher SpO_2_ values in the minutes after birth, particularly the first 3 min, where the median SpO_2_ values were ≥10% higher (Fig. 1). The HR values in DCC infants were stable (median 148–158 throughout the first 10 min), whereas the prior cohort of ICC infants displayed more fluctuation, by starting significantly lower, reaching a higher peak, and then stabilizing (median HR 99 at 1 min, 164 at 5 min, and 157 at 10 min)^48^ (Fig. 2). However, in the Dawson nomogram for vaginally born infants, a proportion of infants born <37 weeks of gestation was included (160/466 infants), which could have resulted in a slightly larger difference between the compared groups.^4^

Björland et al.^49^, recruited a further cohort of 898 term infants born vaginally with DCC of at least 1 min, incorporating the use of a dry electrode ECG device to allow early assessment of HR. More than 50% of infants had HR data available within 15 s after birth. Their findings suggested a rapid increase in HR from median 122 bpm at 5 s to 174 bpm by 60 s, gradually reducing thereafter. The authors suggested that this pattern might have differed from those of previous studies due to underestimation of HR when measured by pulse oximetry.

Newborn infants born at 34–36 completed gestational weeks are defined as late preterm (LPT) and represent approximately 7–8% of all deliveries in high-income countries. The proportion of infants delivered LPT has steadily increased in recent decades.^50^ Compared to term (≥37 completed weeks of gestation) infants, LPT are physiologically immature and have a higher risk of death and/or metabolic, infectious, and respiratory morbidities. Moreover, LPT infants exhibit a tendency towards poorer postnatal adaptation with lower Apgar scores.^51–53^ Consequently, LPT infants frequently need resuscitation, including positive pressure ventilation and oxygen supplementation in the first minutes after birth.^54^

Data from the “healthy” moderate-late preterm population are currently sparse, in part because published work in this group has focused on infants receiving resuscitation, rather than the group that do not require intervention.^55^ One feasibility trial included 44 infants ≥32 weeks’ gestation, of whom 32 did not receive resuscitation during at least 2 min of DCC.^56^ HR was derived from ECG, oximetry, or ultrasound. Outcomes were reported for the entire group of 44 infants, with only two infants with HR < 100 bpm at any point in the first 5 min after birth, and median HR > 160 throughout this time. This trial was the precursor to a recently completed trial that will generate HR and SpO2 data from a larger cohort of infants ≥32 weeks’ gestation (ACTRN12618000621213).

One large RCT conducted by Ashish et al.^57^ has reported outcomes for >000 infants of ≥33 weeks born by uncomplicated vaginal birth, who breathed spontaneously. The majority were born at term (mean 39.4 weeks). Infants were allocated to cord clamping at either <60 s or DCC at ≥180 s, with HR and SpO2 data recorded by a ultrasound transducer and oximetry, respectively. The infants receiving DCC had higher SpO2 values throughout the first 10 min (80% vs 61%, 91% vs 78%, and 98% vs 88% at 1, 5, and 10 min respectively). The HR of the DCC group was lower at 1 and 5 min after birth (107 vs 116 and 132 vs 134 bpm, respectively), but similar thereafter. The mean HR remained between 130 and 140 bpm from 5 to 10 min in both groups of this study, lower than that observed in previous studies such as Smit et al.^45^, where the median HR with DCC was between 146 and 152 in this time. This may reflect differences in setting, method of HR ascertainment, or gestation (term or late preterm), and further data in the late preterm group will be of value.

## Conclusions

In summary, the fetus develops in a lower oxygen environment compared to the newborn infant. However, oxygen provided to fetal tissues is equivalent to that supplying the newborn infant due to metabolic and cardiocirculatory adjustments. Healthy term babies after an uncomplicated birth have a low initial SpO_2_ that rapidly increases, plateauing at values of 90–95% around 3–5 min. Currently available data support the concept that expected ranges for HR and SpO_2_ differ, depending in the timing of cord clamping. Larger cohorts of vaginally born infants indicate that infants receiving DCC experienced a more rapid rise in SpO_2_ than those receiving ICC. HR findings have been less consistent between studies, but it appears that DCC infants may achieve HR stability at an earlier time point after birth than ICC infants. The mode of delivery (cesarean section vs vaginal birth), the mode of HR assessment (ECG vs pulse oximeter), and other factors such as immediate skin-to-skin care may have some influence on measured values. Information regarding SpO_2_ and HR in late preterm infants born after vaginal delivery, and not needing resuscitation, is lacking. Adequately powered prospective studies will provide interesting information on how SpO_2_ and HR evolve during postnatal adaptation in this important cohort of preterm infants.
